# Computational models of ventricular mechanics and adaptation in response to right-ventricular pressure overload

**DOI:** 10.3389/fphys.2022.948936

**Published:** 2022-08-24

**Authors:** Oscar O. Odeigah, Daniela Valdez-Jasso, Samuel T. Wall, Joakim Sundnes

**Affiliations:** ^1^ Simula Research Laboratory, Oslo, Norway; ^2^ Department of Bioengineering, University of California, San Diego, San Diego, CA, United States

**Keywords:** pulmonary hypertension, right ventricle, reduced-order models, finite-element models, growth and remodeling models, ventricular mechanics, ventricular adaptation

## Abstract

Pulmonary arterial hypertension (PAH) is associated with substantial remodeling of the right ventricle (RV), which may at first be compensatory but at a later stage becomes detrimental to RV function and patient survival. Unlike the left ventricle (LV), the RV remains understudied, and with its thin-walled crescent shape, it is often modeled simply as an appendage of the LV. Furthermore, PAH diagnosis is challenging because it often leaves the LV and systemic circulation largely unaffected. Several treatment strategies such as atrial septostomy, right ventricular assist devices (RVADs) or RV resynchronization therapy have been shown to improve RV function and the quality of life in patients with PAH. However, evidence of their long-term efficacy is limited and lung transplantation is still the most effective and curative treatment option. As such, the clinical need for improved diagnosis and treatment of PAH drives a strong need for increased understanding of drivers and mechanisms of RV growth and remodeling (G&R), and more generally for targeted research into RV mechanics pathology. Computational models stand out as a valuable supplement to experimental research, offering detailed analysis of the drivers and consequences of G&R, as well as a virtual test bench for exploring and refining hypotheses of growth mechanisms. In this review we summarize the current efforts towards understanding RV G&R processes using computational approaches such as reduced-order models, three dimensional (3D) finite element (FE) models, and G&R models. In addition to an overview of the relevant literature of RV computational models, we discuss how the models have contributed to increased scientific understanding and to potential clinical treatment of PAH patients.

## 1 Introduction

Pulmonary arterial hypertension (PAH) is clinically described as the sustained rise in pulmonary arterial pressure (PAP) and pulmonary vascular resistance (PVR) due to changes in the pulmonary vasculature. The chronic increase in PAP causes increased afterload on the right ventricle (RV), and results in RV remodeling to enhance cardiac contractile function and maintain cardiac output (Vonk Noordegraaf et al., 2017). The survival in patients with PAH is strongly correlated with the ability of the RV to function under this increased pressure load, while the actual level of pulmonary artery pressure has only minor prognostic significance (Chin et al., 2005). Although it is known that the RV adapts to the increased pressure overload in order to maintain cardiac function, the underlying remodeling mechanisms, as well as the sequence of remodeling processes, are still not well understood (Omens, 1998; Lee L. et al., 2016). Generally speaking, the RV initially increases its wall thickness, stiffness and contractility in response to the sustained pressure overload (Vonk-Noordegraaf et al., 2013). As the disease progresses, the chamber dilates and the shape changes from the typical “crescent” shape to being more spherical, where the interventricular septum bows towards the left ventricle (LV). These structural and functional changes are known to become detrimental to overall cardiac function over time, leading to a progressive reduction of cardiac output, and eventually, right heart failure.

Advances in experimental and clinical techniques have increased our understanding of cardiac diseases, including PAH, as well as opened up more therapeutic avenues to alter the course of detrimental cardiac remodeling. For example, the application of diffusion tensor magnetic resonance imaging (DTMRI), histological analysis, and biaxial mechanical testing has provided insight into how pressure overload can lead to structural remodeling in the RV, in the form of myocardial and extracellular matrix (ECM) stiffening and changes in regional myocardial fiber architecture (Hill et al., 2014; Agger et al., 2016; Vélez-Rendón et al., 2019; Sharifi Kia et al., 2020). Also, through experimental and clinical studies, three separate signaling pathways–the endothelin, nitric oxide and prostacyclin pathways–that contribute to the pathogenesis of PAH have been uncovered. Treatments targeting these pathways can lead to a regression of detrimental cardiac remodeling as well as reducing the morbidity and mortality in PAH patients (Ghofrani et al., 2004; Shao et al., 2011; Lang and Gaine, 2015; Galiè et al., 2016).

In spite of the huge advances in knowledge obtained by experimental and clinical studies, it can be difficult to relate or understand the interdependence of events taking place on different scales. For example, it is challenging to explain how global pump mechanics of the heart chambers relates to the local mechanics of the cardiomyocytes which are responsible for generating the contractile force necessary for the pumping action of the heart. As a result, it becomes equally challenging to understand how changes that occur at the cardiomyocyte level due to pressure overload translate into changes of whole organ form and function. As such, our understanding of PAH and how it leads to remodeling of the RV remains incomplete, despite the wealth of experimental and clinical studies available on the subject. Improved understanding can be gained by complementing these experimental and clinical studies with computational models. Such models not only enable quantitative testing of hypotheses regarding the stimuli for pathological RV remodeling, but can also provide quantitative understanding of the contributions of different remodeling mechanisms such as changes in RV morphology, size and structure, towards compensated or decompensated RV function in PAH. Ultimately, computational models may be able to predict the progression of the disease, and can potentially predict the timeline of the transition from compensatory to decompensatory adaptation, as well as how this timeline can be altered by clinical interventions. Such insight may lead to novel or improved techniques for prognosis, diagnosis and treatment of PAH.

Currently the majority of computational models for studying cardiac physiology and pathophysiology are focused on the LV, and are also limited to snapshots in time, thereby not capturing phenomena such as long-term ventricular adaptation. However, the number of computational studies targeting PAH and the RV has increased in recent years, and there are also examples of models targeting long-term RV adaptation and remodeling. Herein, we review these computational models targeting PAH and RV mechanics, and aim to summarize the main approaches and contributions of the various model categories. Although the main focus will be on computational studies aimed at understanding RV mechanics in PAH, we will include some studies based on other forms of pulmonary hypertension (PH) (Galiè et al., 2016) because they offer valuable scientific insight into overall RV mechanics and adaptation in the setting of pressure overload.

To frame the discussion of computational studies of PAH and its effects on RV mechanics, we first provide a brief description of the current clinical understanding of PAH in Section 2. Computational models of heart mechanics vary greatly in the level of detail as well as conceptual and computational complexity. To organize and facilitate the discussion, we have chosen to categorize the models into three main groups: reduced-order models, three dimensional (3D) models, and growth and remodeling (G&R) models. A more detailed description of these groups is provided in Section 3, followed by a brief review of important contributions within each category. In Section 4 we summarize the main scientific findings from these studies, focusing on insight and understanding of fundamental mechanisms underlying PAH and RV mechanics, while a similar review of clinically significant findings is provided in Section 5. Lastly, we highlight the current challenges and outline possible directions of future approaches that can advance the current state of modeling RV mechanics.

## 2 Current clinical status of PAH

Diagnosis and treatment of PAH have evolved progressively in the past decade, with advances in imaging techniques aiding the early detection and assessment of the disease. However, in spite of these advances the diagnosis of PAH remains a complex process involving clinical expertise in at least cardiology, imaging, and respiratory medicine. The diagnosis is based on a clinical suspicion based on symptoms, patient history, and physical examinations, and includes the review and interpretation of a detailed set of investigations typically referred to as a diagnosis algorithm (Trow and McArdle, 2007; Galiè et al., 2016). The goal of these investigations is to confirm that certain hemodynamic criteria are met and to determine the severity of the disease. Currently, by expert consensus, PAH is defined as a resting mean pulmonary arterial pressure (mPAP) ≥25 mmHg, with a pulmonary arterial wedge pressure (PAWP) ≤ 15 mmHg and a PVR > 3 Wood units (Galiè et al., 2016). Right heart catheterization, an invasive technique for measuring PAP, PAWP and PVR, remains the gold standard for confirming a PAH diagnosis. A non-invasive alternative to right heart catheterization is Doppler echocardiography which can be used to estimate PAP, albeit with less reliability, making it insufficient for PAH diagnosis and for supporting treatment decisions (McLaughlin and McGoon, 2006; Badesch et al., 2009). Other noninvasive techniques for PAH diagnosis include electrocardiography, chest radiography, computed tomography (CT), and cardiac magnetic resonance imaging (cMRI). Detailed descriptions of these diagnostic techniques for PAH (including right heart catheterization) can be found in McGoon et al. (2004) and Trow and McArdle (2007). Although these noninvasive techniques may provide supportive evidence of PAH and aid in patient screening to assess which patients should proceed to invasive cardiac catheterization, they lack sufficient specificity and sensitivity to establish the diagnosis of PAH (Schmidt et al., 1996; Ahearn et al., 2002; McGoon et al., 2004; Alhamad et al., 2011). Ultimately, right heart catheterization is needed for a definitive diagnosis of PAH and to establish that the true basis of elevated PAP is due to diseased pulmonary arteries rather than other causes such as congenital heart defects or left heart disease. There is a clear clinical need to improve noninvasive diagnostic techniques for PAH, which motivates further research into the fundamental disease mechanisms.

The treatment of PAH includes lifestyle modifications as well as conventional and disease-specific treatments, with the goal being to improve RV function and quality of life and to lower the risk of mortality (McLaughlin and McGoon, 2006). In the present review we focus on the disease-specific treatments, and in particular treatments that directly target the RV in PAH, including atrial septostomy (Sandoval et al., 1998), mechanical circulatory support via right ventricular assist devices (RVADs) (Strueber et al., 2009), and RV cardiac resynchronisation therapy (RV-CRT) (Handoko et al., 2010). Some of these treatment strategies for the RV in PAH target the reduction of wall stress, which is a key determinant of RV remodeling in response to pressure overload. For instance, atrial septostomy, which is a surgical procedure that creates an interatrial right-to-left shunt, and mechanical circulatory support using RVADs, target the reduction of RV free wall (RVFW) stress by lowering RV preload. On the other hand, RV-CRT aims to correct the left-to-right ventricular dyssynchrony which is common in PAH patients, and targets the secondary effects of the elevated RVFW stress. For a detailed review on these therapies that directly target the RV in PAH, see Westerhof et al. (2017) and Handoko et al. (2010). An in-depth review of drug-based PAH therapies is found in McLaughlin and McGoon (2006), including diuretic therapy, vasodilators and calcium channel blockers. Despite the development of multiple disease-targeted therapies for severe PAH, lung transplantation remains the most effective and curative treatment (Klepetko et al., 2004; Galiè et al., 2016). Also, therapies targeting the adverse remodeling of the RV are less established (compared to therapies for LV failure (Handoko et al., 2010)) with limited evidence to prove their efficacy (Westerhof et al., 2017). As such, further research is needed before these therapies can be universally recommended in the clinic. However, these therapies have aided in the reduction and delay in patient referral for lung transplantation, because typically, transplantation is reserved for patients with an inadequate clinical response to therapy (McLaughlin and McGoon, 2006; Galiè et al., 2016).

## 3 Multiscale models of RV mechanics and remodeling

**FIGURE 1:**
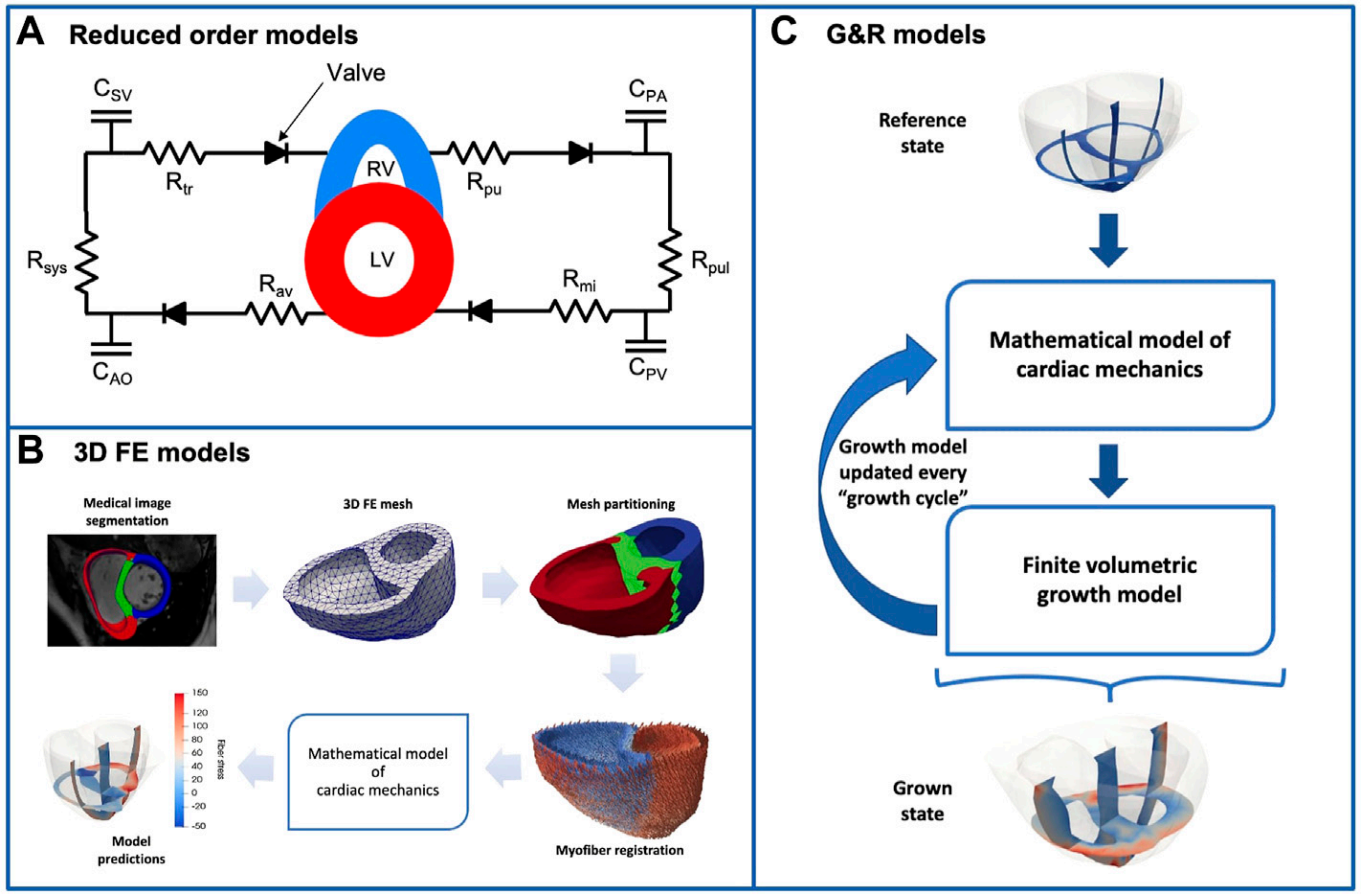
The figure illustrates the three main groups of computational models used in the study of RV mechanics in PAH. **(A)** Reduced-order model representing the whole circulation. The systemic and pulmonary circulations are described by lumped-parameter models, with the LV and RV represented by time-varying elastance chambers or as segments of thick-walled spheres. The parameters and subscripts in the figure have the following interpretation. **(C)** compliance, R: resistance, AO: aorta, SV: systemic veins, PA: pulmonary artery, PV: pulmonary vein, sys: systemic vasculature, pul: pulmonary vasculature, tr: tricuspid valve, pu: pulmonary valve, mi: mitral valve, av: aortic valve **(B)** A 3D FE model of the ventricles. Typically, the ventricular geometry is reconstructed from medical images, and the figure illustrates an example workflow. These 3D FE models include a fairly detailed description of ventricular geometry, myofiber orientation, as well as the passive and active myocardium **(C)** Biventricular G&R model based on the finite volumetric growth theory. These models couple a 3D FE model of the ventricles to a set of equations (called a “growth law”) that describe long-term ventricular adaptation and remodeling as a function of mechanical quantities such as stress and strain.

Computational studies of RV mechanics and remodeling have been carried out using various strategies with varying levels of model complexity, as summarized in Figure 1. One group of studies uses ventricular-vascular coupling models, where the ventricles of the heart are modeled as time-varying elastance chambers coupled to a zero-dimensional model of the vasculature. Such models are useful for studying how increase in afterload on the RV alters the overall mechanics and leads to remodeling of the heart chamber. The complexity of the models varies significantly, both in their representation of the heart and vasculature. In particular, some studies have replaced the time-varying elastance model for the ventricles with thick-walled spherical segments (Arts et al., 2005; Lumens et al., 2009b), or even 3D models (Augustin et al., 2021) which allows a more detailed representation of regional RV mechanics. Regardless of the complexity utilized though, we will collectively refer to this general class of models as *reduced-order models*.

A second class of models takes the more detailed representation of the ventricles one step further, and employs finite element (FE) models to account for the 3D geometry of the ventricles as well as interventricular interaction. As a result, these models give detailed insight into the local distribution of stress and strain in the left ventricular free wall (LVFW), RVFW and septum. They also enable more realistic simulation of overall heart mechanics including ventricular interaction, myofiber mechanics, and the dynamics of the septum. However, these are often stand-alone ventricular models with simplified representations of the vasculature and cardiovascular coupling. In the following discussion, these models are referred to as *three dimensional models*.

As a third class of models, we consider models that go beyond the “snapshots” into cardiac function provided by the models above, and in addition account for the adaptation and remodeling of the RV over time. As such, these models aim to mechanistically describe and predict the adaptation of the RV in response to pressure overload, as opposed to the first two classes of models where the adaptation (such as RVFW thickening or dilation) is generally prescribed in the model rather than predicted by the model. Commonly referred to as *growth and remodeling models*, these models can incorporate changes in mechanical properties resulting from remodeling of the extra-cellular matrix (e.g., fibrosis), in addition to the anatomical adaptations in the form of myocardial growth. Predictive models of G&R can be constructed either as extensions of the 3D models outlined above, or by incorporating a volumetric growth law in the FE models (Rodriguez et al., 1994). It is worth noting that ventricular adaptation has also been simulated using reduced-order models (see, for e.g. (Arts et al., 2005)). However, these studies will be discussed under the *reduced-order models* class. Cardiac G&R models have been developed over several decades, and have been shown to give good general agreement with clinical observations. However, most models only include the LV, and very few of the existing biventricular models explicitly focus on RV G&R. The limitations of existing G&R models will be discussed in more detail below.

### 3.1 Reduced-order models

Reduced-order models give insight into the overall hemodynamics of the cardiovascular system by combining a model of the circulation to a model of the heart. These models typically consist of a lumped-parameter model, open or closed loop, which represents the systemic and pulmonary circulation, connected to a model of the heart chambers. A commonly used reduced-order model is the CircAdapt model (Arts et al., 2005), which simulates beat-to-beat dynamics of the four-chamber heart coupled to the vasculature. The model is also capable of simulating geometric and structural adaptation of the ventricles in response to mechanical load. In the CircAdapt model, the mechanical model of the two ventricles assumes a simplified ventricular geometry where one large outer chamber encapsulates a smaller inner chamber. The inner chamber then represents the LV, while the space between the outer and inner chambers represents the RV. While this approach implicitly includes mechanical interaction between the two ventricles, it does not explicitly describe the septum. This limitation motivated the development of the Three-wall Segment (TriSeg) model of ventricular mechanics (Lumens et al., 2009b). In the TriSeg model - which was incorporated in the CircAdapt model - the LVFW, RVFW and septum are modeled as three thick-walled spherical segments which meet at a common circular junction. This model facilitates more detailed studies of regional mechanics and ventricular interaction, but assumes the myocardial tissue in each segment is homogeneous. In order to allow the modeling of heterogeneity in tissue properties and mechanical behaviour within each segment, the MultiPatch module (Walmsley et al., 2015) was developed as an extension of the TriSeg model.

The TriSeg model coupled to the CircAdapt model or a simplified system model representing the pulmonary and systemic circulation systems has been employed in several computational studies of RV mechanics in PAH. Tewari et al. (Tewari et al., 2013) coupled the TriSeg model to two-element windkessel models (see, e.g., Westerhof et al. (2009)) representing the systemic and pulmonary circulation. To simulate the occlusion of the inferior vena cava (IVC), a technique used experimentally to alter the preload on the RV, the Windkessel model for the systemic circulation was split into two pathways; one representing the anterior circulation that drains into the vena cava, and the other representing the posterior circulation that first drains into the IVC and then into the vena cava. The coupled model was used to investigate how PVR, pulmonary arterial elastance, and pulmonary arterial narrowing changed with PAH progression, by fitting the model to hemodynamic measurements from mice exposed to 0, 14, 21, and 28 days of chronic hypoxia and a vascular endothelial growth factor receptor antagonist, Sugen 5,416 (i.e., SuHx protocol (Taraseviciene-Stewart et al., 2001)) to induce PAH. Philip et al. (2018) and Pewowaruk et al. (2018) used a similar model developed by Tewari et al. (2016b) to investigate how alterations in the structure and mechanics of myocytes due to pressure overload contribute to organ function or dysfunction. The computational framework incorporated a biophysically detailed cardiomyocyte mechanics model (Tewari et al., 2016a) representing cross-bridge kinetics, passive intra- and extracellular passive elastic forces (titin and collagen), maximum myocyte calcium-activated force (*F*_max_), and viscosity, as well as length dependence of the active force. This cardiomyocyte model, which also accounted for the effect of metabolites (such as adenosine triphosphate (ATP), adenosine diphosphate (ADP), and inorganic phosphates) on cross-bridge kinetics, was used to drive the contraction and relaxation of the ventricles in the coupled TriSeg-Windkessel model, and allowed the direct investigation of how cellular level changes impact organ level RV function.

As stated earlier, the CircAdapt model is capable of simulating ventricular adaptation in the form of changes in size and mass. By applying a set of physiological adaptation rules, the model allows the size and mass of the cardiac chambers and blood vessels to adapt so that mechanical tissue load is normalized. Lumens et al. (2009b) tested the capability of their TriSeg model of ventricular mechanics - integrated into CircAdapt - for simulating RVFW hypertrophy in chronic PH. The model of ventricular interaction in CircAdapt is incapable of simulating chronic PH because it presumes that RV pressure is always substantially lower than LV pressure. By replacing this ventricular model with their TriSeg model, and using end-diastolic myofiber stress as the mechanical stimulus for RV adaptation, Lumens *et. al* were able to predict the substantial increase in RVFW thickness (up to 120% compared to a normal heart) in response to increased pulmonary resistance. A similar method was used in two other studies investigating the benefits of early RVFW pacing for improved cardiac function (Lumens et al., 2009a) and the link between early-diastolic LV lengthening and altered RVFW myofiber function (Lumens et al., 2012) both in severe PAH. Despite the focus of both studies on severe decompensated PAH (without tissue adaptation), both studies successfully predicted the increase in RVFW mass in response to mild PAH using the combined TriSeg-CircAdapt model.

There have also been computational studies using Laplace-type reduced-order models to study the effect of RV remodeling as a result of PAH. In these models, the RV is usually approximated as a sphere or fraction of a sphere and the wall is assumed to be thin, which allows the use of Laplace’s law to relate RV wall stress to the blood pressure. Justification for assuming a spherical shape for the RV as opposed to the usual crescent shape arises from previous studies which have shown that the RV becomes spherical in PAH hearts (Valdez-Jasso et al., 2012; Hill et al., 2014). Jang et al. (2017) applied a Laplace-type reduced-order model to study the relationship between myocardial tissue mechanics and hemodynamics in a pressure overloaded RV. The authors presented this relationship using a novel stress-pressure loop which is a plot of the wall stress estimated by the Laplace model against measured RV pressure throughout the cardiac cycle. It is worth noting that Laplace-type models can be used to study RV mechanics during PAH, beyond just the wall stress state. More recently, Vélez-Rendón et al. (2018) and Kwan et al. (2021) employed such a model to discriminate the contributions of morphological and intrinsic mechanisms (i.e., altered contractility and chamber stiffness) towards compensated RV function during the early stages of PAH. Their model of the RV comprised the RV cavity (assumed to be a fraction of a sphere), the RVFW and septum. Although such models are unable to quantify mechanical metrics such as the transmural and regional variations in ventricular stress and strain in PAH, or the dynamics of the septum, they are suitable for investigating scientific or clinical research questions that do not require a complex and detailed computational model of the heart.

### 3.2 Three dimensional models

Most computational studies that use 3D models to investigate altered cardiac mechanics are based on image-derived FE models. Typically, studies that are focused on ventricular mechanics include only the ventricular chambers in the 3D model, with the atria seldom included. The meshes used in these computational studies to represent the heart chambers are either generated from segmentation of medical images or are based on idealized ventricular geometries (i.e., sphere). Unlike reduced-order models, most 3D models do not include a closed loop circulatory description (i.e., arteries and veins) in the modeling framework, and instead rely on simplified representations of these components as hemodynamic boundary conditions for the FE models. Typically, a combination of a windkessel model and prescribed filling pressures is used to describe the dynamic pressure on the endocardial surface, which enters the FE model as a Neumann boundary condition. This is usually supplemented by a combination of Neumann and Robin (i.e., spring-like) boundary conditions on the epicardial surface, and a Dirichlet boundary condition that restrict the motion of the atrioventricular plane. Detailed descriptions of standalone biventricular FE models can be found, for instance, in (Nielsen et al., 1991; Stevens et al., 2003), while a more recent review of patient-specific FE models is found in Sack et al. (2016).

Despite the standalone nature and somewhat artificial boundary conditions that are typically imposed on 3D biventricular models, they are still useful for studying heart remodeling under PAH conditions. In particular, these models can provide detailed insight into the local mechanical state of the RV wall, including how local loads and deformations are affected by increased pressure load as well as altered wall thickness and mechanical properties. A recent computational study by Gomez et al. (2017) employed a 3D biventricular FE model to evaluate the role of the longitudinal myofiber reorientation - which has been shown to occur with the progression of PAH (Park et al., 2016)–as a compensatory mechanism that could lead to improvements in RV function in PAH patients. Avazmohammadi et al. (2019b) developed biventricular FE models for the normal and PAH states, based on data from a rat model of monocrotaline (MCT)-induced PAH (Ghodsi and Will, 1981), to study the link between fiber-level remodeling and variation in wall stress in the post-PAH state. They hypothesized that the increase in *vivo* RVFW stress plays a crucial role in the remodeling of the myofibers. However, still very little is known of the structural alterations of the RV myocardium in response to PAH, and how these changes relate to RV function. A structural-based constitutive model for the normal RV myocardium, which directly accounts for the contributions of myo- and collagen fibers as well as their interactions, has been developed (Avazmohammadi et al., 2017b). The authors later extended this model to account for tissue-level changes experimentally observed in hypertensive tissue such as increased collagen fiber recruitment. However, as at the time of writing this review, this constitutive model is yet to be incorporated into an organ-level FE model for *in silico* assessment of RV adaptation in PAH.

Other authors have employed 3D FE models to study the effect a pressure overloaded RV has on ventricular interaction and mechanics. For example, Xi et al. (2016) developed patient-specific biventricular FE models of a normal subject and PAH patient to study the effect of the disease on RV and LV mechanics, and also the relationship between septal curvature and the transseptal pressure gradient in PAH patients. A more recent study conducted by Kheyfets et al. (2019) used an FE model of the human heart ventricles (reconstructed from MR data) to study the effect of increased RV rigidity and RV myocardial fiber re-orientation - both structural adaptations known to occur in the RVFW in response to PAH (Hill et al., 2014) - on LV torsion mechanics. Along similar lines, Finsberg et al. (2019) developed a patient-specific biventricular FE model to evaluate the changes in regional (i.e, RVFW, LVFW and septal) contractility associated with PAH progression and to uncover a noninvasive clinical index useful for assessing PAH severity in patients.

Some computational studies have used 3D FE models coupled to reduced-order models of the vasculature to study RV adaptation in PAH. For example Shavik et al. (2019) used a patient-specific 3D FE model coupled to a two-element Windkessel model of the systemic and pulmonary circulation to study the effects of RVADs on biventricular mechanics in the context of PAH. Scardulla et al. (2018) used a similar modeling strategy to show the potential of *in silico* modeling for non-invasive assessment of RV hemodynamics and for the prediction of RV mechanics in patients with PH. In both studies, the parameters of the coupled FE-Windkessel model were calibrated to match the patient-specific hemodynamic measurements.

### 3.3 Growth and remodeling models

The modeling efforts reviewed so far can provide detailed insight into the mechanical state of the RV during PH, which is valuable for understanding and predicting how the RV adapts and remodels over time. However, the natural next step in modeling is to move beyond the snapshots consisting of one or a few heart cycles, and derive models that describe long-term adaptation and remodeling. Such models have been developed for several decades, and are capable of describing both morphological changes in the form of ventricular hypertrophy and dilation, and structural changes such as fibrosis, which affect the mechanical properties of the tissue. However, most of the developed models have been focused on the LV, and models of RV G&R remain relatively rare. Depending on the scientific or clinical questions, the availability of anatomical data or the required speed of computations, the model for the heart chambers can be two-dimensional thick-walled spherical segments (already discussed in Section 3.1) or detailed 3D FE models (Kerckhoffs et al., 2012). In this section, we will review the latter class of models used in computational studies of RV adaptation in response to PAH.

Most existing 3D models of G&R are based on the volumetric growth framework proposed by Rodriguez et al. (Rodriguez et al., 1994). This framework describes volumetric growth of elastic soft tissues by a multiplicative decomposition of the observed growth deformation gradient into a growth component and an elastic component (see, for instance, Holzapfel (2001) for a detailed introduction to soft tissue mechanics modeling, including the role of the deformation gradient). The deformation induced by growth is not necessarily compatible, as it may induce discontinuities and overlapping tissue volumes, but the inclusion of the elastic deformation ensures an overall deformation which is compatible and consistent with the applied boundary conditions. The formulation provides a general framework suitable for describing morphological changes as well as residual stress arising from growth. Other formulations exist, for instance based on mixture theory (Humphrey and Rajagopal, 2002; Ambrosi et al., 2010), but these have not yet been applied in studies of ventricular G&R. The volumetric growth and constrained mixture theory only provide the general frameworks for describing soft tissue growth, and they need to be complemented with constitutive laws describing how mechanical factors trigger G&R. For the case of the volumetric growth framework, such laws typically prescribe the growth tensor *F*_*g*_ as a function of local stress and/or strain. Detailed reviews and perspectives on computational models of G&R can be found in (Ambrosi et al., 2011; Menzel and Kuhl, 2012), while (Witzenburg and Holmes, 2017) provides an interesting comparison of phenomenological growth laws for describing myocardial hypertrophy. A phenomenological growth law capable of describing the clinically relevant *reverse remodeling* was proposed by Lee et al. (Lee et al., 2015). Growth laws that rigorously capture the underlying biology and tissue microstructure are still relatively rare, as discussed in the recent reviews by Niestrawska et al. (Niestrawska et al., 2020) and Sharifi et al. (Sharifi et al., 2021).

Several authors have studied G&R of cardiac tissue, mostly focused on the LV and based on either stress or strain as the biomechanical stimuli for cardiac growth. Kerckhoffs et al. (2012) applied a strain-based growth law in a biventricular FE model coupled to a closed-loop reduced-order model of the circulation to test if a single growth law could reproduce different modes of LV hypertrophy. The coupled model was used to predict concentric hypertrophy (in response to pressure overload due to aortic stenosis) and eccentric hypertrophy (in response to volume overload due to mitral valve regurgitation). These results led the authors to conclude that strain (rather than stress) could be the driving biomechanical stimuli for cardiac G&R. A more recent study by Lee L. C. et al. (2016) studied local G&R in response to a myocardial infarction. The authors also applied a strain-based growth law capable of predicting both cardiac G&R and reverse growth when the loading is reduced. The growth law was integrated in an FE model of the LV and used to predict eccentric hypertrophy due to volume overload caused by a non-contractile infarct. The model was also used to predict reverse growth as the stiffness of the infarct was increased - a technique that has been proposed in experimental studies to attenuate post-myocardial infarction remodeling (Mukherjee et al., 2008; Morita et al., 2011). Rausch et al. (2011) applied a stress-based growth model to describe pressure-overload induced G&R of both the LV and RV. The growth model was integrated in a biventricular FE model to predict concentric hypertrophy of the LV and RV due to systemic and pulmonary hypertension respectively. In addition, their results suggested that LV hypertrophy was highly localized - with no significant effect on RV shape and size - whereas RV hypertrophy led to a change in LV shape (from circular to D-shaped) as a result of the flattening of the septum. A similar study by Genet et al. (2016) builds on the work by Rausch et al. (2011) and describes both pressure- and volume-overload induced G&R. However, in contrast to Rausch et al. (2011), they considered a strain-based stimulus for growth and used a four-chamber heart model enabling the study of secondary effects of ventricular hypertrophy such as papillary muscle displacement, outflow obstruction, valve annulus dilation, and regurgitant flow. Avazmohammadi et al. (2019a) combined biventricular FE models of rat hearts with a strain-driven volumetric growth law and a model for fiber adaptation that would drive the fiber orientation towards the direction of maximum stretch. The G&R model showed good agreement with experimental data, and highlighted the important contribution of the fiber reorientation to the RV mechanical adaptation.

## 4 Scientific insights

In early-stage PAH, compensated RV function is largely maintained by a combination of geometric remodeling in the form of hypertrophy and material remodeling in the form of increased contractility (Vonk Noordegraaf et al., 2017). To distinguish the roles of morphological and material property changes on the preservation of RV function in a rat model of MCT-induced PAH, Vélez-Rendón et al. (2018) employed a Laplace-type reduced-order model to show that in the early stage of the disease (i.e., up to 2 weeks post-MCT administration), hypertrophy and increased myocardial contractility contributed equally to the preservation of RV stroke volume and function in the presence of elevated PAP. In fact, both mechanisms contributed equally to the preservation of stroke volume into the late stage of the disease which corresponds to 4 weeks post-MCT administration. Altered myocardial stiffness, on the other hand, was a compensatory mechanism only at 1 week post-PAH induction, where their model results showed a decrease in the stiffness of the RVFW. This result indicated that the transient decrease in diastolic stiffness was necessary to preserve the stroke volume before the onset of hypertrophy and augmented contractility in subsequent weeks. As the disease progressed, diastolic myocardial stiffness increased, which was associated with reduced cardiac function, consistent with the findings of earlier experimental studies by Rain et al. (2013) and Trip et al. (2015).

Myocardial structure and mechanics are altered in response to pressure overload as shown in animal experimental models of PAH (Bogaard et al., 2009b; Rain et al., 2013; Vélez-Rendón et al., 2019; Kwan et al., 2021). The alteration in structure is mainly in the form of myocardial fibrosis, which leads to increased RV diastolic stiffness, supplemented by altered RV contractile force (Rain et al., 2016; Wang et al., 2018). These changes at the cellular and molecular level contribute to RV compensation in early-stage PAH and subsequent RV failure in chronic PAH (Bogaard et al., 2009b). However, the direct impact of these cellular and molecular level changes on organ level function is still not very clear. In a study by Philip et al. (2018), a multi-scale modeling approach was used to investigate the combined effects of cellular level changes and pressure overload on RV function in a mouse model of bleomycin-induced RV failure (Thrall et al., 1979). The structural and mechanical changes modeled on the cell level were myocardial fibrosis and impaired myocyte maximum force generation (*F*_max_), which are both characteristic of bleomycin-induced PH (Wang et al., 2010), and both of these were investigated in combination with RV pressure overload. They found that pressure overload alone led to a decrease in ejection fraction but preserved cardiac output, while a combination of pressure overload and myocardial fibrosis did not show any significant impact on RV function, beyond pressure overload alone. However, when considering decreased *F*_max_ in combination with pressure overload and fibrosis, the results showed a decrease in both ejection fraction and cardiac output, suggesting that impaired myocyte maximum force generation has a strong impact on RV organ level function, and could be a marker of the transition from RV dysfunction with preserved cardiac output to RV failure due to PH.

A similar study investigating the link between cellular and tissue level changes and RV function in PH was carried out by Pewowaruk et al. (2018). Employing the same computational framework used by Philip *et al.*, the authors investigated the relative importance of changes in metabolite concentrations (i.e., ATP, ADP and inorganic phosphates), *F*_max_ and RV myofiber stiffness on RV function in three mouse models of PH: 1) PAH induced by the Sugen Hypoxia protocol (SuHx) (Taraseviciene-Stewart et al., 2001); 2) bleomycin-induced PH (Bleo) (Thrall et al., 1979); and 3) PH secondary to left heart disease (PH-LHD) caused by a myocardial infarction. They also investigated if the relative effect of altered metabolite concentrations and *F*_max_ on RV function was dependent on the degree of RV afterload. When considered in combination with increased RV afterload, their results showed that changes in passive stiffness of the RVFW had little effect on cardiac output (which was the metric used to assess overall RV function) for both the SuHx and Bleo models. They did not perform simulations of altered myofiber stiffness for the PH-LHD model since this phenomenon has not been reported for this model of PH. On the other hand, alterations in cell-level metabolite concentrations and tissue-level *F*_max_ led to a high degree of ventricular-vascular uncoupling (for all three models of PH) and decreased cardiac output (for the SuHx and Bleo models of PH), suggesting that these changes at the cell and tissue level were critical to RV function in the setting of pressure overload. In other words, their simulations suggested that the RV was able to maintain cardiac output in response to pressure overload alone, and that changes in RV mechanoenergetics (i.e., metabolite concentrations and myocyte maximum force generation) were required to cause a decrease in cardiac output. However, the relative effect of these changes in mechanoenergetics were dependent on the degree of RV afterload. For lower levels of afterload - one to two times increase in PVR - changes in *F*_max_ had a stronger correlation with RV ejection fraction compared to altered metabolite concentrations. On the other hand, at higher levels of afterload - four times increase in PVR–both changes had similar correlations with RV ejection fraction.

Experimental studies have documented that the pressure overloaded RV can influence LV and septal mechanics, in particular the diastolic filling of the LV (Bemis et al., 1974; Schena et al., 1996; Marcus et al., 2001; Puwanant et al., 2010; Hardegree et al., 2013). In fact, chronic RV pressure overload has also been shown to lead to electrophysiological remodeling of the LV by causing a prolongation of the action potentials, effective refractory periods and a slowing of the longitudinal conduction velocity (Hardziyenka et al., 2012). This influence is most likely mediated by ventricular interdependence because the RV and LV do not function in isolation (Belenkie et al., 2001). In a study to investigate the significance of chronic RV pressure overload on LV function, Schena et al. (1996) showed that chronic RV pressure overload impaired LV diastolic function albeit without any systolic impairment. In another noninvasive study of the echocardiographic images acquired from 44 patients with evidence of PH, Puwanant et al. (2010) showed that chronic RV pressure overload influences the geometry of the LV and septum, which in turn impairs LV torsion.

To provide some mechanistic insight, Kheyfets et al. (2019) used a realistic FE model of a pediatric heart to elucidate the inverse relationship between LV torsion, apical rotation and RV remodeling in PAH. In other words, as the degree of RV structural remodeling increased, there was a noticeable decrease in LV torsion and apical rotation during contraction. They observed an exponential relation between RV pressure and LV myocardial stress, and speculated that the decrease in LV torsion/torsion rate could be a result of this myocardial stress increase. As it is known that gene and protein expression is sensitive to mechanical stress (Lowes et al., 1997; Friehs et al., 2013), the authors suggested that these changes in interventricular mechanics in PAH, which lead to increased LV myocardial stress, could drive changes in LV fiber orientation and gene/protein expression, as has been observed in the hypertensive RV (Lowes et al., 1997). However, it is worth noting that from their simulations, Kheyfets et al. (2019) observed that these changes in LV mechanics due to RV adaptation in PAH had minimal impact on the ejection fraction of the LV. This observation is in contrast to an earlier study by Xi et al. (2016), also based on a human heart model, which suggested that PAH can lead to LV remodeling as well as significantly reduced ejection fraction for both the RV and LV. Xi et al. (2016) however stated that these results should be extrapolated with caution given they were based on only one computational model of PAH. Furthermore, Xi *et al.* also showed that passive stiffness, contractility and myofiber stress were all substantially increased in both the RV and LV of the PAH patient compared to the normal subject. In addition, their simulations revealed an approximately linear relationship between the curvature of the septum and the systolic transseptal pressure gradient. They concluded that this relationship suggests that septal curvature has the potential to serve as a non-invasive marker for quantifying the pressure state in the RV in PAH patients.

Histological studies have revealed that the pressure overload leads to a re-organization of local myocyte orientation (Hill et al., 2014; Avazmohammadi et al., 2017b). In particular, there is a reorientation of the myocytes towards the longitudinal direction, but the link between this tissue-level remodeling and RV function in the setting of pressure overload remains poorly understood. Avazmohammadi et al. (2019b) developed 3D FE rat heart models to provide some insight into the link between myocyte reorientation and the wall stress state in a rat model of MCT-induced PAH. Their simulations showed that the reorientation of the myocytes towards the longitudinal direction correlated strongly with the elevated longitudinal stress in the post-PAH rat heart. Furthermore, transmural wall stress distribution in the RVFW of the post-PAH rat showed a stronger variation compared to that in the control rat which was essentially uniform across the RVFW. While the circumferential stress in the RVFW of the post-PAH rat was nearly restored to homeostatic values, the longitudinal stress was much higher in the post-PAH rat compared to the control rat, consistent with earlier computational studies by Avazmohammadi et al. (2017a) and Jang et al. (2017) which used Laplace-type reduced-order models based on experiments of pulmonary artery banding (PAB) induced PH in rats (Faber et al., 2006). The simulations by (Avazmohammadi et al., 2019b) indicated that the largest values of longitudinal stress in the post-PAH rat were recorded in the midwall region of the RVFW, suggesting a transmural variation in the passive material properties of the RVFW in PAH. This transmural variation has been suggested to be a result of denser and more fibrous connective tissue in the midwall region of a hypertensive RVFW (Avazmohammadi et al., 2017a). Their model predictions showed that the transmural changes in RVFW stiffness - with a concentration in the midwall region–caused a considerable increase in RV end-diastolic volume EDV or preload, leading to an increase in RV stroke volume and ejection fraction. They suggested that this transmural variation in passive stiffness could serve as a secondary tissue-level remodeling mechanism that facilitates RV dilation in severe PAH, with the primary mechanism being the lengthening of the cardiomyoctes. In addition, they examined the independent effect of pressure overload on RV function by simulating pressure overload on a normal heart, without any of the anatomical or structural adaptations in a PAH heart. The results indicated that the normal RV could preserve its ejection fraction with much lower increase in RVFW contractility compared to a PAH heart, suggesting that other maladaptive changes (such as myocardial fibrosis and possibly myocyte reorientation) increase the need for a higher contractility of the RVFW to cope with the increased pressure overload. In other words, pressure overload alone was not sufficient to cause RV dysfunction, but pressure load in combination with known anatomical and structural changes during PAH was, which is consistent with the findings of other computational studies by Philip et al. (2018) and Pewowaruk et al. (2018).

As reviewed in Section 3.3 above, there are relatively few examples of direct applications of G&R computational models to the RV and PH. Although a number of computational studies have been conducted, models of cardiac G&R are still in their infancy, and several fundamental questions remain unresolved. For instance, growth laws using stress (Rausch et al., 2011) and strain (Genet et al., 2016) have reproduced the same growth phenomena, leaving the exact role of these two potential mechanical drivers unclear. The detailed comparison of existing growth laws presented in Witzenburg and Holmes (2017) revealed interesting differences between the models’ ability to reproduce known growth patterns, which could guide further development of models. Alternative model frameworks based on constrained mixture theory Humphrey and Rajagopal (2002) have been suggested to overcome some of the known limitations of the volumetric growth framework Yoshida and Holmes (2021). Combined with a shift from phenomenological towards more mechanistic growth laws, this more complex framework may shed light on the most important growth stimuli and potentially contribute to resolving some of the fundamental questions of the field. Examples of such largely unresolved questions include, for instance, what mechanical stimuli are the main drivers of pathological growth, how they are sensed and processed in the cells, and whether we can delineate the role each of these processes play towards preserved RV functionality as the disease progresses. Advances in computational (and experimental) research into PH can provide answers to such questions, which will substantially improve understanding of the disease, and by extension, guide the design of therapeutic strategies geared towards improved clinical outcomes in PH patients.

## 5 Clinical applications

In this section, we will highlight the findings of computational studies focusing on clinical aspects of PH-induced RV dysfunction. These studies have mainly targeted one of two distinct goals. The first is to unravel new and improved clinical biomarkers of PH-induced RV dysfunction using *in silico* modeling techniques. These studies are important because of known limitations of existing non-invasive biomarkers as highlighted in Section 2. The second is to investigate the effect of clinical strategies that have been proposed to reverse or stop adverse RV remodeling. These latter studies have thus added to the body of work necessary to achieve confidence in these treatment methods. In particular, we will focus on three common therapies that have been suggested to be effective in improving cardiac performance in PAH patients: atrial septostomy (AS), implantation of RVADs and RV-CRT via RV pacing.

In patients with chronic PAH, the synchronous contraction and subsequent relaxation of the LV and RV is lost (López-Candales et al., 2005; Marcus et al., 2008). This ventricular dyssynchrony manifests in the shortening of the RVFW beyond pulmonary valve closure, and the lengthening of the LV and septum immediately after aortic valve closure. Lumens et al. (2012) suggested that the dyssynchrony can serve as a marker of PH-induced RV dysfunction because experimental studies have shown it is strongly correlated with the severity of the disease (López-Candales et al., 2005; Kalogeropoulos et al., 2008). In particular, they hypothesized that the lengthening of the LVFW during early-diastole was a result of altered RVFW myofiber load. Using the CircAdapt model to simulate PH with complete, incomplete and no structural RV adaptation to increased myofiber load, they were able to show that LV early-diastolic lengthening - assessed using the LV early-diastolic strain index (LVEDSI) - progressively increased with increasing contractile dysfunction of the RVFW. In other words, there was an increase in early-diastolic LVFW lengthening as RV adaptation to PH decreased. They concluded that increased LVEDSI reflects inadequate structural adaptation of the RVFW to increased myofiber load, and can therefore be a useful and non-invasive marker of PH-induced RV dysfunction.

Another study by Palau-Caballero et al. (2017) investigated the link between ventricular dyssynchrony and rapid leftward septal motion (RLSM) which is the leftward bowing of the septum during early LV diastole in patients with chronic PAH (López-Candales, 2015). This phenomenon, which has been observed experimentally, appears to indicate a worsening state of RV function and thus can also serve as a marker of RV failure with PAH progression (López-Candales, 2015). RLSM has been suggested to be a result of a negative transseptal pressure gradient (i.e., negative LV-RV end-diastolic pressure gradient) due to increased RV afterload (Tanaka et al., 1980; Kingma et al., 1983). Using the CircAdapt model, Palau-Caballero *et al.* tested this hypothesis and also attempted to unravel other mechanisms that might be responsible for RLSM. They simulated three different stages of PAH; mild, moderate, and severe PAH (corresponding to mPAP of 40, 60, and 80 mmHg, respectively). The simulations showed pronounced RLSM for both moderate and severe PAH, but a negative transseptal pressure gradient was only prevalent in the severe PAH simulations, suggesting that increased RV afterload due to PAH is not the only mechanism responsible for RLSM. As their results showed that the prolonged RV shortening in PAH causes dyssynchrony in ventricular relaxation which in turn causes RLSM, they concluded that the change in septal motion in PAH patients is induced both by altered RV afterload and ventricular relaxation dyssynchrony. In addition, their model simulations showed that PAH in combination with LV hypotrophy (degeneration of the LV due to cell loss) resulted in decreased RLSM, while PAH in combination with RV hypotrophy resulted in increased RLSM.

In order to obtain a conclusive diagnosis of PAH and for guiding subsequent patient therapy, right heart catheterization is required (Montani et al., 2013). As stated in Section 2, there are non-invasive techniques that can be used for PAH diagnosis such as Doppler echocardiography and cMRI (Bouchard et al., 1985; Chan et al., 1987). However, due to the low sensitivity of these non-invasive testing methods, inconsistency in the measurement techniques as well as frequently reported inaccuracies in the estimation of pulmonary artery pressure using Doppler echocardiography, right heart catheterization remains the gold standard (Celermajer and Marwick, 2008; Hsu et al., 2008; Fisher et al., 2009; Constantinescu et al., 2013). Scardulla et al. (2018) hypothesized that using a closed-loop model of the cardiovascular system comprising a lumped-parameter model of the circulation and a patient-specific biventricular FE model of the heart can provide a computational alternative for extracting both hemodynamic and biomechanical parameters relevant to PAH diagnosis. Their goal was to ascertain the ability of their computational approach to noninvasively assess any impairments on RV function and also to predict RV mechanics and ventricular interactions in patients with PH. The model predictions showed good agreement with clinically measured parameters such as stroke volume, cardiac output, cardiac index, end-systolic elastance (a load-independent measure of RV contractility), arterial elastance, and lastly RV-arterial coupling. Furthermore, the model reproduced the PH-associated motion of the septum, which was in good agreement with septal motion measured by cMRI, and showed elevated myocardial fiber stress throughout the cardiac cycle in patients with PH compared to healthy control patients. These model results suggests that there is potential for extracting biomechanical markers of RV failure (which are currently only extracted invasively in the clinic) noninvasively using a computational approach.

Finsberg et al. (2019) also unraveled a potential non-invasive marker of RV remodeling in response to PAH using patient-specific models for healthy control and patients diagnosed with PAH. They reconstructed the patient-specific geometries from cMRI images and also fitted their computational models to clinical data comprising pressure and volume waveforms as well as regional circumferential strains in order to estimate the contractility of the myocardium. Their numerical experiments suggested that there is a mechanical basis for using the EDV ratio (RVEDV/LVEDV) as an indicator of the degree of RV remodeling in PAH patients, because they found that this ratio varied substantially in the PAH group but minimally in the control group. In other words, the EDV ratio can serve as a clinical index to delineate between PAH patients with mild RV remodeling (RVEDV/LVEDV <1.5 ) and those with severe RV remodeling (RVEDV/LVEDV >1.5 ). In addition, their results showed that for patients with mild RV remodeling, peak RVFW contractility was not significantly different from that in the control group. However, for patients with severe RV remodeling, peak RVFW contractility was significantly reduced (up to 25%) compared with the control group. As such, the EDV ratio can (by extension) also serve as a clinical index for estimating RVFW contractility in PAH patients.

RV-CRT has been proposed as a therapeutic strategy to correct ventricular relaxation dyssynchrony that occurs in advanced stages of PAH (Handoko et al., 2009; Westerhof et al., 2017). As described above, the relaxation of the RV in severely decompensated PAH patients is delayed due to the increased workload on the myofibers in the RVFW, leading to a dyssynchronous interventricular contraction-relaxation pattern. As such, the goal of RV resynchronization therapy is to stimulate the RV via RV pacing in order to unload the RVFW early enough to maintain ventricular synchrony during relaxation. To investigate the efficacy of this therapy, Lumens et al. (2009a) used the TriSeg model coupled to the CircAdapt model of the circulation to simulate ventricular mechanics under both normal and PAH conditions. They were able to show that in severe PAH conditions, early RVFW pacing may improve ventricular synchrony by decreasing the myofiber load on the RV, thus leading to a homogenization of the load over both ventricular walls. This workload homogenization thus leads to improvements of RV pump function.

AS has been shown to result in clinical and hemodynamic improvements leading to an improved long-term survival of patients with severe PH (Kerstein et al., 1995; Kurzyna et al., 2007; Law et al., 2007; Sandoval et al., 2011). Theoretically, the creation of this interatrial communication should allow for the decompression of a hypertensive RV and preservation of systemic cardiac output via an augmentation of systemic blood flow (Austen et al., 1964), but the actual mechanisms by which the process benefits patients are poorly understood. A computational study to shed light on these mechanisms was conducted by Koeken et al. (2012), using the CircAdapt model of the cardiovascular system to attempt to uncover the mechanism through which AS offers symptomatic and hemodynamic improvements in PH patients. Their simulations suggested that AS only improved cardiac dynamics in severe cases of PH because it led to a net right-to-left shunt flow, which increased LV preload and thereby helped in maintaining systemic arterial pressure. In patients with mild PAH, AS caused a left-to-right shunt flow resulting in a further increase of RVFW workload and exacerbated symptoms in the patients. Contrary to findings in previous studies (Kerstein et al., 1995; Rothman et al., 1999), their simulations suggested that the relief of symptoms and improvement in exercise capacity seen in severe PH patients after AS cannot be explained by an increase in oxygen delivery to the peripheral tissues. They found that there was no increase in the oxygen available to the peripheral tissue as a result of AS because the right-to-left shunt flow caused by the atrial septal defect (ASD) led to a mixing of oxygen-poor blood and oxygen-rich blood in the left atrium. Also, venous oxygen saturation was not increased as a result of the ASD, suggesting that the tissue had not been exposed to higher oxygen levels. Rather, they concluded that the beneficial effects of AS can be explained by the improvement in systemic blood flow which facilitated the maintenance of systemic arterial pressure during exercise in severe PH patients. These findings were consistent with an earlier computational study by Diller et al. (2010) which showed that although arterial delivery of oxygen increased as a result of AS, the average state of tissue oxygenation remained unchanged, implying that improved oxygen delivery is not responsible for the improvement in systemic cardiac output observed in severe PAH patients following AS.

Mechanical circulatory support for the RV, using implantable RVADs, has been considered as a treatment strategy for PAH. The idea is to unload the RV in diastole, thereby lowering the pressure (and by extension, the stress) on the RVFW, and effectively increasing cardiac output (Krabatsch et al., 2011; Verbelen et al., 2015). *In silico* experiments have been conducted to gain insight into the efficacy of this therapy in improving the cardiac output of patients with severe PAH, and also the long-term hemodynamic effects (on the ventricles and vasculature) of such mechanical support. Punnoose et al. (2012) used a reduced-order model to investigate the theoretical effects of an RVAD on aortic, pulmonary arterial, ventricular and atrial pressure waveforms in a PAH patient. The RVAD was modeled as a continuous flow pump with inflow sourced from either the RV or right atrium (RA) and outflow to the pulmonary artery. Their results suggested that RVAD support was capable of increasing cardiac output by decreasing RV end-diastolic pressure and volume and increasing LV filling and arterial pressure. These improvements were noted to decrease with increasing PAH severity due to the progressive increase in PVR. In addition, their simulations showed that RVAD support led to detrimental increases in mPAP and pulmonary capillary pressure. However, they showed that these potentially adverse effects of RVAD support could be mitigated by operating RVADs at low flow rates, which was supported by the findings of an experimental study by Verbelen et al. (2015). The low flow strategy is necessary so as to avoid high blood flows through the pulmonary bed which already has high vascular resistance in severe PAH patients. The findings from Punnoose *et al.* were supported in a recent in-silico study by Shavik et al. (2019) which employed a similar RVAD model, albeit with a more detailed 3D model for the ventricles coupled to a lumped-parameter model of the vasculature. The authors concluded that in order for RVAD support to be an effective therapy for end-stage PAH patients, the implantation and operation speed of the device needs to be optimized depending on the severity of the disease.

## 6 General challenges and future directions

As noted above, there is a strong correlation between the RV function and survival in patients with PAH (Vonk-Noordegraaf et al., 2013), implying that there is a clear clinical need to study and understand how the RV adapts to this disease. As we have discussed in this review, computational models can aid in understanding G&R on multiple levels. Most of the existing models offer only a snapshot of the mechanical state of the RV, and are biventricular, limiting their ability to the study of only primary effects of RV overload such as wall thickening/dilation and altered tissue properties. Although these models can still be immensely useful for understanding the drivers of RV adaptation, their utility can be increased by incorporating the atrial chambers in the modeling framework, to allow the study of secondary effects of RV overload such as papillary muscle dislocation or tricuspid annular dilation leading to regurgitant flow (Kim et al., 2006; Rogers and Bolling, 2009). There are a few four-chamber models of heart mechanics and electro-mechanics available in the literature (Fritz et al., 2014; Land and Niederer, 2018; Pfaller et al., 2019; Strocchi et al., 2020, 2021; Gerach et al., 2021). In the future, these models can be adapted to study both the primary and secondary effects of RV overload. Alternative frameworks based on reduced-order models are able to predict global RV adaptation to PAH, but offer limited insight into the local mechanical drivers and consequences of RV G&R. The most comprehensive models, which are still relatively few, combine detailed cardiac mechanics with growth models in order to predict local and regional G&R of the RV over time.

While all the models reviewed here hold substantial potential for scientific and clinical applications, there is a paucity of experimental and clinical data to parameterize the computational models, which limits their current clinical relevance. It is worth noting that sensitivity analysis techniques such as Morris screening method (Morris, 1991) and Akaike information criterion (AIC) (Burnham, 1998) have been used previously to access which parameters in a cardiac computational model can be uniquely identified given sparse data (Gerringer et al., 2018; Guan et al., 2019; van Osta et al., 2020). In other words, these techniques help to determine the smallest subset of parameters required in a computational model to still be able to describe and predict the available clinical and experimental data. A different approach to mechanical parameter estimation was taken by (Li et al., 2020), who applied optimal experimental design to maximize the information gained from mechanical tests. These techniques should all be explored and developed further to improve the characterization of RV mechanics.

**FIGURE 2:**
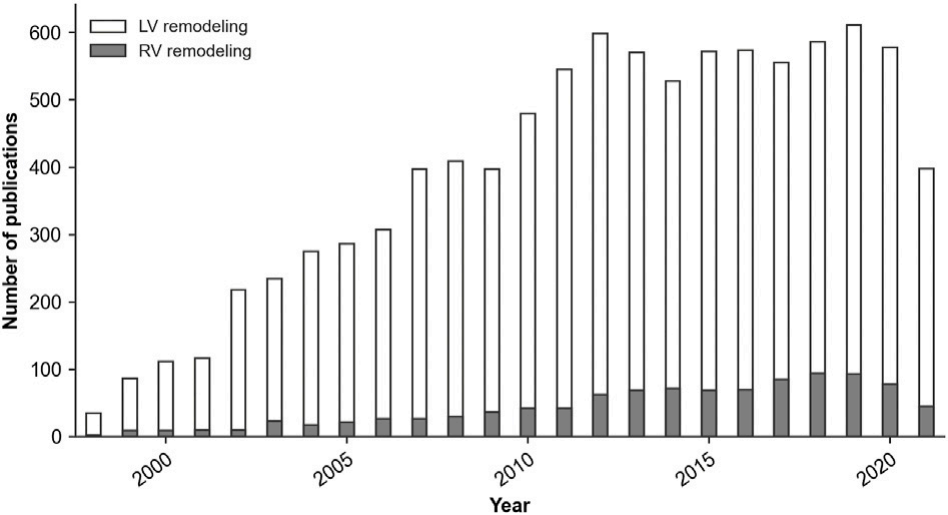
Number of yearly publications of peer-reviewed articles with ‘Left ventricular remodeling’ or ‘Right ventricular remodeling’ as the major topic of the publication. *Source*: PubMed^®^ Medical Subject Headings (MeSH) database, March 2022.

The lack of specific RV data is partly linked to the relatively low experimental and clinical focus on the RV, as illustrated in Figure 2, and partly to challenges of imaging, data acquisition, and modeling, caused by the relatively thin wall and complex geometry. As such, continued development and advancement of computational models in this field should be accompanied by a generally increased research attention to the RV, and in particular by increased experimental and clinical research into PAH mechanisms and pathological G&R of the RV.

While computational models of cardiovascular mechanics have been developed over several decades, and are very well established, models of cardiac G&R must be considered an emerging field. Most such models are based on the volumetric growth theory briefly described in Section 3.3, and rely on a constitutive relation which describes the growth of the tissue as a function of the local mechanical state. Such growth laws are by nature phenomenological, and are derived based on experimental or clinical observations of macroscopic changes in tissue size and shape. While this phenomenological and rule-based nature leads to conceptually simple models with relatively low computational cost, the models are mechanobiologically limited in that they are not based on the underlying growth processes that lead to changes in volume and composition of the tissue. Alternative model frameworks exist, with the *constrained mixture theory* being a particularly relevant example, see, e.g., Humphrey and Rajagopal (2002). These models enable tracking the composition of the tissue over time, by considering the deposition and removal of its individual constituents, and may, for instance, offer detailed insight into the drivers and effects of ECM remodeling. However, the additional biological detail comes at the cost of a considerable increase in computational complexity compared with the volumetric growth framework. At present, the literature includes several examples of constrained mixture models describing G&R of arteries, e.g., Gleason et al. (2004); Gleason and Humphrey (2004); Baek et al. (2006); Karšaj et al. (2010), but no examples of 3D constrained mixture models of cardiac growth.

A final limitation worth mentioning is that current models of cardiac G&R in PAH only consider local mechanical drivers of growth in the form of stress and strain. While these are known to be key drivers of cardiac G&R, the process is also strongly influenced by hormonal signals (Bogaard et al., 2009a; Voelkel et al., 2012). Although current models which incorporate only the mechanical signaling pathways have proven successful at predicting the different patterns of growth such as cardiomyocyte thickening and lengthening, they are limited in their ability to predict clinical outcomes from pharmacological interventions which typically target the hormonal signaling pathways. As a result, there is a need to build computational models that incorporate both altered mechanical load and hormone signal pathways in PAH in order to better understand the processes that trigger pathological RV G&R. Such models could also help to elucidate the link between biological factors at the molecular and cellular level, and biomechanical factors at the organ and systemic level. Such multiscale models will also enable the in-silico testing of various drug therapies designed to improve the quality of life of PAH patients. An example of a multiscale G&R model that incorporates both mechanical and hormonal signaling pathways is presented in the work by Estrada et al. (2021). Albeit focused on the LV, future adaptations of such a model can be applied to study G&R of the RV.

In summary, the results and findings highlighted in this review show that computational models have already made significant contributions to our understanding of RV mechanics and adaptation in PAH. As stated in Section 4, important scientific contributions include the isolation of individual disease mechanisms and components, in order to assess their relative contribution and importance for RV function and remodeling. Clinical contributions, as reviewed in Section 5, include novel noninvasive biomarkers for PAH, which may potentially replace or supplement existing invasive diagnostic tools. However, the potential of computational models is far from fully utilized, and the scientific and clinical findings should mainly be interpreted as promising directions of research rather than conclusive results. Future progress relies on further refinement of models as well as close interaction between experimental, clinical, and computational researchers. Experimental data is critical for model parameterization and validation, and insight gained from computational models may guide future experiments as well as clinical studies. Models can provide detailed insight into the mechanical consequences of PAH, which in turn may generate insight and hypotheses on how these mechanical alterations drive adaptation and pathological remodeling of the RV. Furthermore, fundamental questions such as the relative role of anatomical growth versus tissue remodeling, e.g., wall thickening versus altered stiffness and contractility, are highly amenable for study with computational models. While computational models of cardiac mechanics have been developed for several decades, the majority of models have focused on the LV. However, over the last 10–15 years we have witnessed a shift of community interest towards detailed biventricular models. This development has led to an increasing availability of advanced models and computational tools, which significantly advances the potential for computational modeling of RV mechanics and PAH. In terms of modeling long-term G&R, further model refinement should focus on developing general growth laws that capture a range of different scenarios, as well as delineating the scope and utility of phenomenological versus the more complex and biologically based approaches. Continued and coordinated effort of computational and experimental scientists will unravel important scientific questions about cardiac G&R, and hold the potential to greatly advance the general understanding of PAH and its clinical management.
